# Phylogeographic analysis reveals an ancient East African origin of human herpes simplex virus 2 dispersal out-of-Africa

**DOI:** 10.1038/s41467-022-33214-y

**Published:** 2022-09-17

**Authors:** Jennifer L. Havens, Sébastien Calvignac-Spencer, Kevin Merkel, Sonia Burrel, David Boutolleau, Joel O. Wertheim

**Affiliations:** 1 Bioinformatics and Systems Biology Graduate Program, University of California San Diego, La Jolla, CA USA; 2 Viral Evolution, Robert Koch Institute, Berlin, Germany; 3 Virology Department, National Reference Center for Herperviruses (Associated Laboratory), AP-HP-Sorbonne University, Pitié-Salpêtrière Hospital, Paris, France; 4 Sorbonne University, INSERM UMR-S 1136, Pierre Louis Institute of Epidemiology and Public Health (IPLESP), Paris, France; 5 Department of Medicine, University of California San Diego, La Jolla, CA USA

**Keywords:** Phylogeny, Evolution, Herpes virus, Population dynamics

## Abstract

Human herpes simplex virus 2 (HSV-2) is a ubiquitous, slowly evolving DNA virus. HSV-2 has two primary lineages, one found in West and Central Africa and the other found worldwide. Competing hypotheses have been proposed to explain how HSV-2 migrated out-of-Africa (i)HSV-2 followed human migration out-of-Africa 50-100 thousand years ago, or (ii)HSV-2 migrated via the trans-Atlantic slave trade 150-500 years ago. Limited geographic sampling and lack of molecular clock signal has precluded robust comparison. Here, we analyze newly sequenced HSV-2 genomes from Africa to resolve geography and timing of divergence events within HSV-2. Phylogeographic analysis consistently places the ancestor of worldwide dispersal in East Africa, though molecular clock is too slow to be detected using available data. Rates 4.2 × 10^−8^−5.6 × 10^−8^ substitutions/site/year, consistent with previous age estimates, suggest a worldwide dispersal 22-29 thousand years ago. Thus, HSV-2 likely migrated with humans from East Africa and dispersed after the Last Glacial Maximum.

## Abstract (web summary)

There are competing hypotheses for human herpes simplex virus 2’s migration out-of-Africa. Here, the authors sequence 65 new herpes simplex virus 2 genomes with a focus on under-sampled sub-Saharan African countries, suggesting an Eastern African origin for global dispersal the virus between 22-29 thousand years ago.

## Introduction

Human herpes simplex viruses 1 and 2 (HSV-1, HSV-2) are globally ubiquitous human pathogens. HSV-1 and HSV-2 are slow-evolving double-stranded DNA viruses that can infect via oral and genital routes, though HSV-1 is predominantly associated with oral herpes and HSV-2 with genital herpes^1^. Although genital herpes infection is mostly mild, HSV-2 infection can be fatal in neonates and it is associated with an increased risk of HIV acquisition^2,3^. In 2016, worldwide the prevalence of HSV-2 was 13%; the highest prevalence is in Africa with around 44% of females and 25% of males living with HSV-2^4^. HSV-2 has ancient zoonotic origins, whereby proto-humans in Africa were infected by an ape simplexvirus^5–7^.

Evolutionarily, HSV-2 is comprised of two major phylogenetic lineages separated by a long internal branch. One of these lineages is predominantly found in African populations (which we refer to here as lineage A), and the other lineage which includes members sampled across the world (which we refer to here as lineage B)^8^. The relationship between these lineages suggests HSV-2 diversified in the human population in Africa before dispersing with modern humans out-of-Africa^8^, sometime between 50 and 100 kya^9,10^. Alternatively, it has been hypothesized that HSV-2 left Africa far more recently, sometime between 1671 and 1792 CE, via the trans-Atlantic slave trade^11^ which was active between 1501–1867 CE^12^. Phylogeographic analysis of other viruses suggests that the slave trade may have been a conduit for viral introduction into the Americas^13^.

The inability to distinguish between these two competing hypotheses operating on vastly different time scales arises due to a lack of strong temporal signal in the HSV-2 molecular clock when calibrated using viral sampling dates (i.e., tip dating calibration)^14^. There is no detectable relationship between the year of HSV-2 sampling and the distance from the root of the phylogeny^11,15^. The human migration out-of-Africa hypothesis for HSV-2 dispersal arises from molecular clock estimates calibrated based on inference of codivergence events deeper in the simplexvirus tree that suggest a genome-wide clock rate on the order of 10^−8^ substitutions/site/year^5,7,8,16^. In contrast, the trans-Atlantic slave trade hypothesis is based upon a power-law rate decay of the molecular clock, whereby more recent branches have a faster clock rate than older branches^17^. The absence of a molecular clock calibrated exclusively within HSV-2 leaves these competing hypotheses unresolved.

Importantly, each of these origin hypotheses is associated with different geographic sources within Africa. If HSV-2 accompanied the earliest human migration out-of-Africa, then the most recent common ancestor (MRCA) of the worldwide diversity in lineage B would emanate from East, Central, or Southern Africa, where the first modern human migration began^10^. However, if HSV-2 left Africa by the trans-Atlantic slave trade, then this lineage would originate from West or Central Africa, where most Africans enslaved via the trans-Atlantic slave trade originated. Currently, most available African HSV-2 genomes are from East Africa^8,11,18^, limiting our ability to formally test these hypotheses.

Here, we clarify the location of the emergence of HSV-2 out-of-Africa by sequencing 65 HSV-2 genomes from 18 previously unsampled countries, including a majority of viral samples from West and Central Africa. We further narrow down the timing of the worldwide dispersal through simulation and empirical molecular clock analysis to determine which origin scenarios are compatible with a lack of clock-like signal in HSV-2. We show that HSV-2 lineage B followed human migration from East Africa, but likely postdated the initial out-of-Africa human migration event.

## Results

### HSV-2 genomes

**Fig. 1:**
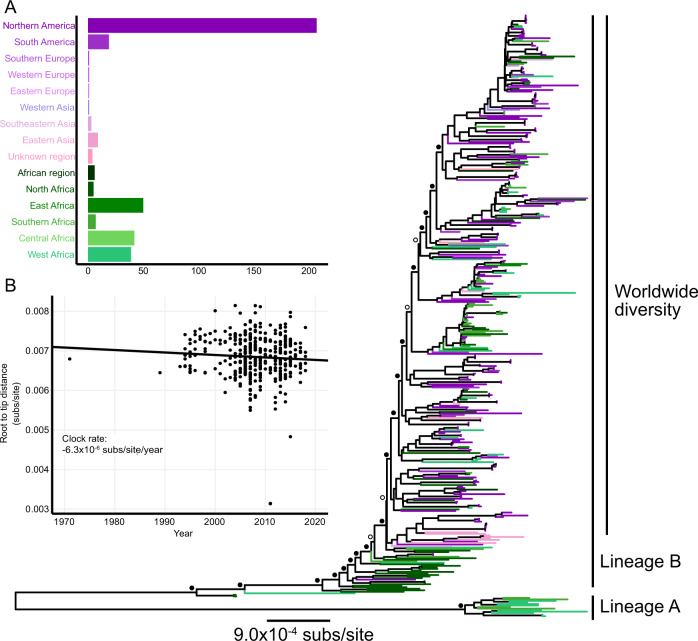
Maximum likelihood phylogenetic analysis of empirical HSV-2 genomes. **A** HSV-2 dataset of 395 sequences by number of samples from each region. **B** Maximum likelihood phylogenetic tree inferred under a GTR + F + R4 model. Tip color indicates geographic region of sampling. The tree is midpoint rooted, though the identical rooting orientation is recovered using the closely related chimpanzee simplexvirus (ChHV). Filled circles indicate nodes with aBayes support >0.95; open circles indicate nodes with aBayes support <0.95, support values in Supplementary Data 2. **C** Root-to-tip distance in maximum likelihood tree versus sampling year with an inferred clock rate of −6.3 × 10^−6^ substitutions/site/year.

We sequenced the enriched DNA of 65 new HSV-2 isolates, including 47 with a plausible link to 13 African countries, permitting us to determine sequence across a mean of 77% of the HSV-2 genome (range: 45-91%). These 65 newly sequenced African HSV-2 genomes were combined with previously published HSV-2 genomes resulting in an alignment of 395 taxa, sampled over 47 years from 37 countries (Supplementary Data 1; Fig. 1A). The total alignment length was 105,520 nt and recombinant fragments were masked.

### Maximum likelihood phylogenetic inference

We inferred a maximum likelihood phylogeny using IQTree2^19^, which supported the existence of two distinct HSV-2 lineages (Fig. 1B). These lineages are separated by a relatively long internal branch with length of 0.009 substitutions/site. The nodes defining lineage A and lineage B both had strong topological support (aBayes = 1.0; Supplementary Data 2). Both midpoint rooting and outgroup rooting using the chimpanzee herpesvirus (ChHV) supports rooting the HSV-2 tree on this long branch, consistent with previous studies^8,11^. Lineage A comprised sequences from West Africa (*n* = 9) and Central Africa (*n* = 4).

Lineage B contains 382 sequences from all sampled geographic regions, including from West and Central Africa. The basal sequences of this lineage are primarily East African. The basal structure of lineage B has strong phylogenetic support (aBayes 0.95–1.00 for the first 8 nodes; Fig. 1). The crown of lineage B, representing the worldwide diversity, has little observable geographic structure (Fig. 1B) due to the oversampling of genomes from North America and limited sampling of other regions^11,20,21^.

### Clock rate inference of empirical sequences

In an attempt to estimate the timing of divergence events within HSV-2, we performed molecular clock analysis using TreeTime^22^. However, despite the inclusion of HSV-2 genomes sampled over a 47-year timespan, we inferred a negative clock rate (Fig. 1C) and a coefficient of determination (*R*^2^) of the regression between year of sampling and root-to-tip distance of 0.00. These findings indicate a lack of clock-like signal, consistent with previous investigations of the HSV-2 molecular clock using a tip-dating approach^11,15^.

We first explored the possibility that this lack of signal was due either to the phylogenetically recessed branches basal to the worldwide diversity in lineage B or the long branch separating the two lineages. However, after excluding the recessed basal taxa, we still failed to detect clock-like signal. Similarly, we failed to find of clock-like signal including only the taxa associated with worldwide diversity within lineage B (Supplementary Fig. 1) or only lineage A. These sensitivity analyses all produced coefficient of determination for the molecular clock of *R*^2^ < 0.1.

### Likelihood of empirical clock rates

**Fig. 2:**
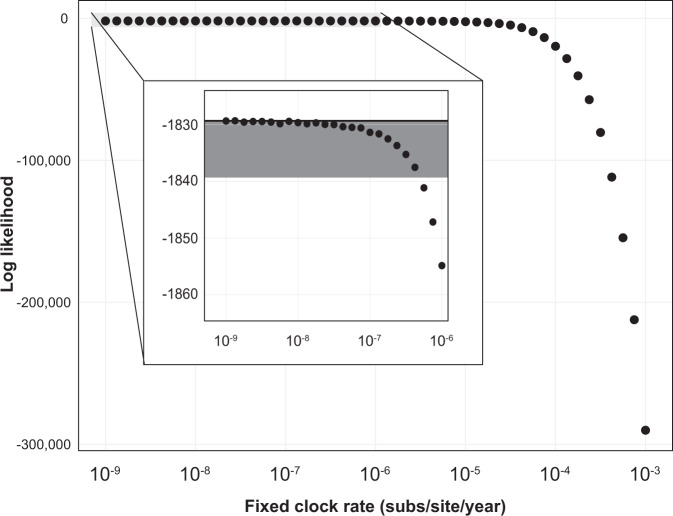
Shape of likelihood surface for HSV-2 clock rate on empirical data. Log-likelihood of HSV-2 chronogram with a range of fixed clock rates estimated using the maximum likelihood topology. The black line is best-estimated log-likelihood. The gray shaded box is the area shown in insert. Within the insert, the black line is maximum estimated likelihood at rate of 10^−9^ substitutions/site/year, and the dark gray box highlights the range 10 points below the maximum estimated log likelihood and encompasses the rates 4.2 × 10^−7^ substitutions/site/year and slower.

To understand the plausible range of evolutionary rates that would be compatible with the lack of clock-like signal in the empirical HSV-2 data, we explored the shape of likelihood surface across a series of fixed substitution rates ranging between 10^−9^ and 10^−3^ substitutions/site/year to determine which molecular clock rates applied to the input ML tree have the highest likelihood and what rates are substantially worse. Given that the maximum likelihood rate estimate is negative, we expected that the slowest substitution rates (i.e., closest to a negative rate) should have the best likelihood. We restricted this analysis to rates between 10^−9^ and 10^−3^ substitutions/site/year, as this range encompasses previously estimated rates for a variety of viruses. As expected, the slowest rates have the best likelihood scores and the fastest rates have the worst (Fig. 2). Clock rates from 10^−9^ through 4.2 × 10^−7^ substitutions/site/year are within the 10 log-likelihood points of the best likelihood. Substitution rates faster than 4.2 × 10^−7^ substitutions/site/year are a substantially worse fit to the observed data.

We note when we performed the same procedure using a virus with strong molecular clock signal, Ebolavirus from 2014 to 2015 West African epidemic^23^, the peak of this log-likelihood curve reasonably approximated the inferred maximum likelihood rate (Supplementary Fig. 2).

### Estimating clock rate of simulated data

To explore at what substitution rates temporal signal should theoretically be detectable in our HSV-2 dataset, given empirical sampling dates and phylogenetic structure, we simulated HSV-2 genome sequences across the maximum likelihood tree under a series of substitution rates, ranging between 10^−9^ and 10^−3^ substitutions/site/year. We then inferred maximum likelihood tree branch lengths from these simulated data and re-inferred the clock rate. A simulated rate that had detectable temporal signal using this approach would be inconsistent with the empirical data.

**Fig. 3:**
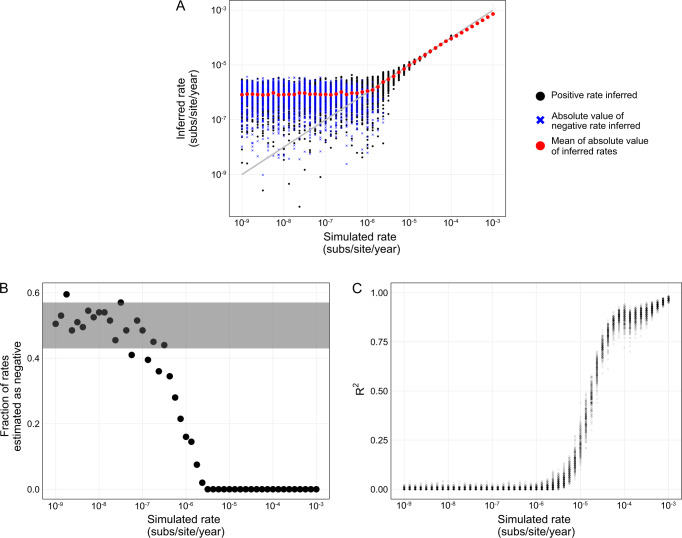
Performance of clock rate inference on simulated data. **A** Inference of clock rate for a fixed input tree with simulated sequences and empirical dates. Black dot represents estimates of positive rates, a blue x represents the absolute value of estimates of negative rates, and red dots indicate the mean of absolute value for each fixed rate. Gray line indicates parity of simulated and estimated rate. **B** Negative clock rate analysis of the fraction of total simulations for a fixed rate that have a rate estimated as negative. Gray shading represents range for which a negative estimate is equally probable as positive (two-sided binomial test *p* < 0.05). **C** R^2^ coefficient of determination of root-to-tip regression, a transparent dot represents one replicate. 200 replicates of simulated sequences were generated under GTR + F + Γ_4_ model then clock rate estimated on simulated sequences; code to produce the simulation results is at https://github.com/jennifer-bio/HSV2.

We were able to reliably infer clock rates from sequences simulated at 1.0 × 10^−6^ substitutions/site/year to 1.0 × 10^−4^ substitutions/site/year (Fig. 3A), indicating the true underlying HSV-2 substitution rate must be slower than these rates. We note that rates faster than 10^−4^ substitutions/site/year were underestimated as the age of the root approached the earliest sample date. Based on a breakpoint analysis, for simulated rates slower than 1.0 × 10^−6^ substitutions/site/year, the mean of the absolute value of inferred clock rates did not capture the simulated rate, unlike faster rates (segmented regression; Supplementary Fig. 3), suggesting these slower rates are consistent with the empirical data.

As in the empirical analysis, the estimated clock rates simulated under slower rates are often negative (Fig. 3B). Thus, sequences simulated under a clock rate which results in an estimated negative rate indicate that the true data could have been produced under that rate. The fastest rate at which more than 5% of trials infer a negative rate on the simulated data is 1.8 × 10^-6^ substitutions/site/year (Fig. 3B). The fastest rate at which it is equally probable for a negative rate to be inferred as positive rate is 3.2 × 10^−7^ substitutions/site/year (binomial test; *p* < 0.05).

The *R*^2^ of the root-to-tip by sample date regression decreases with slower simulated clock rates (Fig. 3C), because as the clock rate (slope) approaches zero less of the divergence is explained by sampling date. More than half of the *R*^2^ estimates were zero in simulations with a rate of 1.8 × 10^-6^ substitutions/site/year or slower (Fig. 3C). The simulations of slower rates are consistent with the root-to-tip regression of the empirical HSV-2 dataset, which had an *R*^2^ of zero.

### Timing the ancestor of HSV-2

**Fig. 4:**
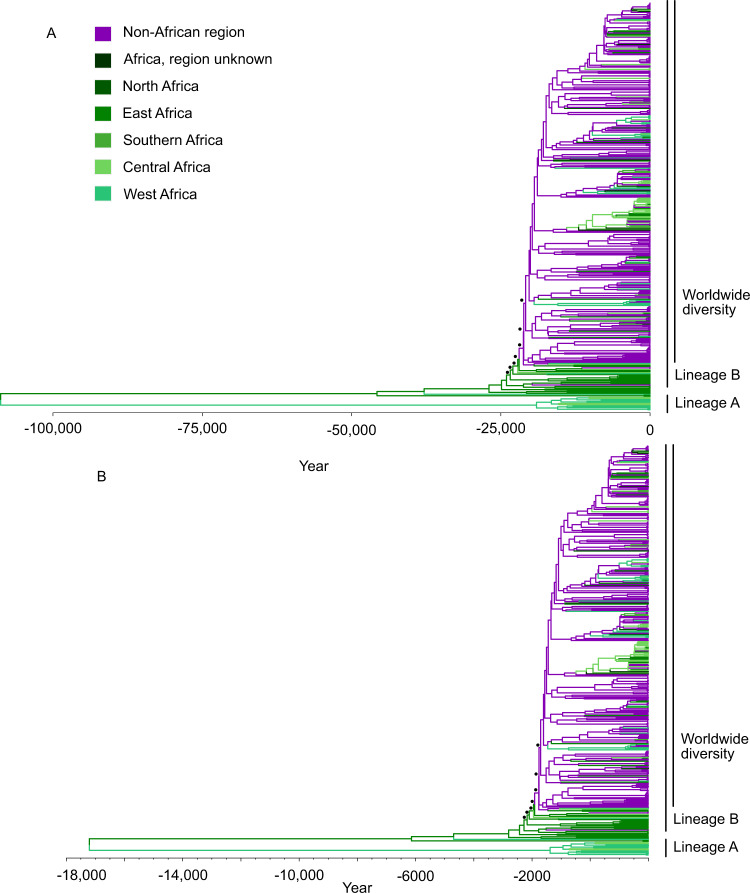
Phylogeography of HSV-2 supports East African origin of out-of-Africa dispersal. **A** Time tree inferred using the evolutionary rate of 5.6 × 10^−8^ substitutions/site/year. **B** Time tree inferred with the fastest rate supported by all clock analyses: 3.2 × 10^−7^ substitutions/site/year. Maximum likelihood tree topology scaled to time tree, colored by plurality of support in migration analysis for each region. Time scale represents the calendar year; negative years are BCE. Black dots indicate nodes used in out-of-Africa weighted age calculation (see Supplementary Fig. 1).

The range of substitution rates we explored is broad, accounting for scenarios in which the time of most recent common ancestor (tMRCA), or root age, for HSV-2 is older than modern humans, 6 million years ago at 10^−9^ substitutions/site/year, to the similarly unrealistic 49 years ago at 10^−3^ substitutions/site/year (Table 1, Supplementary Data 3). The youngest tMRCA not rejected by any of our clock-rate simulation was 19 kya, assuming 3.2 × 10^−7^ substitutions/site/year (Fig. 4B). Using the slowest substitution rate that could be detected in our simulations, 2.4 × 10^−6^ substitutions/site/year, we infer root age of 2.6 kya (556 BCE). Therefore, the HSV-2 tMRCA must be older than this date in order to be consistent with the lack of clock-like signal in the empirical data (Supplementary Fig. 4). HSV-2 tMRCAs between 109 and 146 kya, which are consistent with a previously characterized East-West split in anatomically modern humans in Africa around 110–160 kya^10^, were obtained assuming a substitution rate between 4.2 × 10^−8^ and 5.6 × 10^−8^ substitutions/site/year, respectively (Fig. 4A). These tMRCAs are also consistent with previously published HSV-2 root age 90–120 kya, based on externally calibrated molecular clocks^7,8^.

**Table 1:** Age of root and out-of-Africa dispersal across clock rates

Substitution rate^a^	Root age^b^	Out-of-Africa age^b^	Support for East African source^c^	Molecular clock analyses^d^
1.0 × 10^−9^	6.1 mya	1.2 mya	0.977	Not rejected
1.3 × 10^−9^	4.6 mya	922 kya	0.977	Not rejected
1.8 × 10^−9^	3.4 mya	693 kya	0.977	Not rejected
2.4 × 10^−9^	2.6 mya	519 kya	0.977	Not rejected
3.2 × 10^−9^	1.9 mya	387 kya	0.977	Not rejected
4.2 × 10^−9^	1.4 mya	290 kya	0.977	Not rejected
5.6 × 10^−9^	1.1 mya	219 kya	0.977	Not rejected
7.5 × 10^−9^	809 kya	163 kya	0.977	Not rejected
1.0 × 10^−8^	616 kya	124 kya	0.976	Not rejected
1.3 × 10^−8^	455 kya	93 kya	0.977	Not rejected
1.8 × 10^−8^	341 kya	70 kya	0.976	Not rejected
2.4 × 10^−8^	256 kya	52 kya	0.977	Not rejected
3.2 × 10^−8^	192 kya	39 kya	0.977	Not rejected
4.2 × 10^−8^	146 kya	29 kya	0.977	Not rejected
5.6 × 10^−8^	109 kya	22 kya	0.977	Not rejected
7.5 × 10^−8^	81 kya	16 kya	0.977	Not rejected
1.0 × 10^−7^	61 kya	12 kya	0.977	Not rejected
1.3 × 10^−7^	45 kya	9.3 kya	0.977	Not rejected
1.8 × 10^−7^	34 kya	6.9 kya	0.977	Not rejected
2.4 × 10^−7^	26 kya	5.2 kya	0.978	Not rejected
3.2 × 10^−7^	19 kya	3.9 kya	0.977	Not rejected
4.2 × 10^−7^	14 kya	2.9 kya	0.977	Rejected by some
5.6 × 10^−7^	11 kya	2.2 kya	0.976	Rejected by some
7.5 × 10^−7^	8.1 kya	1.7 kya	0.977	Rejected by some
1.0 × 10^−6^	6.1 kya	1.2 kya	0.976	Rejected by some
1.3 × 10^−6^	4.6 kya	935 ya	0.976	Rejected by some
1.8 × 10^−6^	3.4 kya	702 ya	0.976	Rejected by some
2.4 × 10^−6^	2.6 kya	528 ya	0.976	Rejected by all
3.2 × 10^−6^	1.9 kya	401 ya	0.975	Rejected by all
4.2 × 10^−6^	1.4 kya	303 ya	0.976	Rejected by all
5.6 × 10^−6^	1.1 kya	230 ya	0.977	Rejected by all
7.5 × 10^−6^	826 ya	175 ya	0.977	Rejected by all
1.0 × 10^−5^	616 ya	133 ya	0.980	Rejected by all
1.3 × 10^−5^	463 ya	104 ya	0.982	Rejected by all
1.8 × 10^−5^	353 ya	80 ya	0.985	Rejected by all
2.4 × 10^−5^	267 ya	64 ya	0.989	Rejected by all
3.2 × 10^−5^	205 ya	55 ya	0.990	Rejected by all
4.2 × 10^−5^	159 ya	52 ya	0.980	Rejected by all
5.6 × 10^−5^	125 ya	50 ya	0.944	Rejected by all
7.5 × 10^−5^	101 ya	49 ya	0.827	Rejected by all
1.0 × 10^−4^	84 ya	49 ya	0.540	Rejected by all
1.3 × 10^−4^	72 ya	48 ya	0.274	Rejected by all
1.8 × 10^−4^	64 ya	48 ya	0.129	Rejected by all
2.4 × 10^−4^	59 ya	48 ya	0.062	Rejected by all
3.2 × 10^−4^	55 ya	48 ya	0.039	Rejected by all
4.2 × 10^−4^	53 ya	47 ya	0.026	Rejected by all
5.6 × 10^−4^	51 ya	47 ya	0.020	Rejected by all
7.5 × 10^−4^	50 ya	47 ya	0.013	Rejected by all
1.0 × 10^−3^	49 ya	47 ya	0.010	Rejected by all

^a^Substitutions/site/year.

^b^Prior to most recently sampled virus, 2018.

^c^Maximum inferred probability for East African ancestor prior to out-of-Africa dispersal.

^d^Empirical and simulation analyses described in Figs. 2 and 3.

### Phylogeographic analysis indicates East African origin of worldwide lineage

We performed phylogeographic reconstruction in TreeTime on all empirical time trees inferred with fixed clock rates between 10^−9^ and 10^−3^ substitutions/site/year to determine the ancestral location of HSV-2 and the source of out-of-Africa migration. Using the substitution rates with HSV-2 tMRCA that is consistent with human divergence and published HSV-2 ages (4.2 × 10^−8^ and 5.6 × 10^−8^ substitutions/site/year), we estimate that the ancestor of lineage A was in West Africa and that the out-of-Africa dispersal event within lineage B occurred in East Africa between 29.3 to 21.9 kya (Fig. 4; Table 1).

The phylogeographic placement of East Africa as the source of the HSV-2 out-of-Africa migration is robust across substitution rates (Table 1). Support for East Africa as the ancestral location of lineage B increases with slower substitution rates, exceeding 0.95 for all evolutionary rates that were not rejected by the molecular clock analyses (i.e., <2.4 × 10^−6^ substitutions/site/year). Even the youngest possible estimate for the timing of HSV-2 out-of-Africa migration that is consistent the lack of clock-like signal, is 3.9 kya (1883 BCE) and supports East Africa as the source of the out-of-Africa migration (Table 1). The out-of-Africa age estimate that is consistent with our most permissive rejection strategy was 702 years ago (1316 CE), still predating the trans-Atlantic slave trade by 200 years. Nonetheless, even at this younger age, the out-of-Africa dispersal was still inferred to be in East Africa. Furthermore, the inference of East African origin for the worldwide diversity is robust to geographic partitioning schemes of African and non-African regions (Supplementary Data 4).

## Discussion

**Fig. 5:**
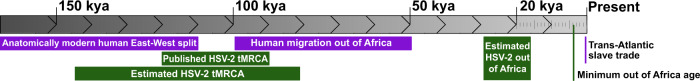
Timeline of inferred HSV-2 diversification events with context of human activity. Purple segments denote events associated with human activity. Green segments denote viral evolutionary events. Timing of the deepest anatomically modern human split is inferred to be 110–160 kya^10^. Timing of first human migrations out of Africa is estimated to be 50–100 kya^10^. Timing of trans-Atlantic slave trade ca. 1501–1867 CE^12^. Previously published age of HSV-2 tMRCA is estimated to be 90–120 kya^7,8^). Age of HSV-2 tMRCA at fixed rates 4.2 × 10^−8^–7.5 × 10^−8^ substitutions/site/year. Age of extant out-of-Africa dispersal estimated with fixed rates 4.2 × 10^-8^–7.5 × 10^−8^ substitutions/site/year, is 16–29 kya. Most recent age of out-of-Africa dispersal estimated under clock rate that is consistent with molecular clock analysis is 3.9 kya (3.2 × 10^−7^ substitutions/site/year).

Understanding the geographic spread of human pathogens, both ancient and recent, provides insight into what conditions lead to viral establishment or extinction, informing the potential dynamics of future epidemics^24–26^. To understand the geography and timing of HSV-2 dispersal, we expanded the geographic breadth of HSV-2 genomic sampling and established the sensitivity of tip-dated molecular clock inference. Clock rates between 4.2 × 10^−8^–5.6 × 10^−8^ substitutions/site/year, are consistent with our molecular clock analysis of empirical data and, under these rates we infer an HSV-2 tMRCA of 110–146 kya which is consistent with the previously published timing between 90 and 120 kya^7,8^. Based on these rates, we conclude that HSV-2 most likely accompanied humans migrating from East Africa and dispersed worldwide between 22 and 29 kya (Fig. 5). The tMRCA of modern worldwide diversity, between 22 and 29 kya, is consistent with an HSV-2 strain dispersing after the population bottleneck associated with the Last Glacial Maximum^27^. This strain could have been the first HSV-2 to disperse worldwide, or it could have replaced HSV-2 that accompanied human migrations and later went extinct (as has been reported in HSV-1)^28^.

HSV-2 has ancient zoonotic origins, whereby proto-humans in Africa were infected by an ape simplexvirus^5–7^. While the tMRCA of HSV-2, the split of lineage A and B, may represent the age of HSV-2 in humans this divergence may have occurred sometime after establishing in humans. The divergence within HSV-2 may reflect human migration within Africa, however, as HSV-2 likely infected humans the tMRCA almost certainly postdates the origin of modern humans. Therefore it is unlikely that the true substitution rate is slower than 2.4 × 10^-8^ substitutions/site/year, which produce a of 250 kya which is older than evidence of anatomically modern humans^10^. Thus HSV-2 migrating with modern humans during the original out-of-Africa migration between 50 and 100 kya^10^, is not consistent with the age of HSV-2 worldwide diversity.

We conclude that a scenario in which HSV-2 left Africa via the trans-Atlantic slave trade between 1500 and 1850 CE is inconsistent with both molecular clock and phylogeographic inference. Out-of-Africa migrations events more recently than ca. 1300 CE would necessitate a viral substitution rate that would produce some clock-like signal using tip-date calibrated molecular clock (i.e., faster than 1.8 × 10^−6^ substitutions/site/year). Our clock-signal breakpoint analysis (Supplementary Fig. 3) indicates that an out-of-Africa event post-dating 1083 CE (i.e., substitution rate 1.3 × 10^−6^ substitutions/site/year) would necessitate a molecular clock that could be inferred from the dataset. Further, any internally consistent molecular clock rate places the origin of this out-of-Africa migration in East Africa, on the opposite side of the continent from where the majority of Africans enslaved as part of the trans-Atlantic slave trade originated. We acknowledge that it is possible that HSV-2 was subsequently imported into the Americas via the trans-Atlantic slave trade long after the worldwide dispersal, given that there is evidence of other viral pathogens being transported to the Americas from Africa during this time^13^. Nonetheless, we find that both the timing and geographical source of the HSV-2 worldwide dispersal are incompatible with the slave trade hypothesis.

A possible explanation for the previous inference of a young age of the HSV-2 out-of-Africa dispersal^11^ is the misapplication of substitution rate correction for purifying selection. As viruses evolve for long periods of time, purifying selection can lead to underestimation of branch lengths due to substitution saturation^29,30^. In RNA viruses, this effect is most prominent on internal branches >0.1 substitutions/site^5,30^, which is an order of magnitude longer than the total tree height of HSV-2 (0.0083 substitutions/site). Bayesian approaches have been developed to explore deeper divergence events in virus phylogenies^5,31^. Additionally, rescaling branch lengths by a power law decay rate can be used to correct for the underestimation^17^. We note that a refinement of the formulation for the power law decay rate model^32^ now describes rate variation through time with a sigmoid curve, acknowledging a more biologically plausible constant evolutionary rate near the tips of viral evolutionary trees with rate decay occurring only deeper in the tree. In other words, although rate decay is almost certainly relevant to deeper divergence events within the simplexvirus phylogeny that show evidence of saturation^5^, HSV-2 is young enough that traditional maximum likelihood phylogenetic inference would not underestimate branch lengths, and applying a power law decay rate would inappropriately underestimate the evolutionary time along recent branches.

Previous analysis of HSV-2 geographic structure has primarily partitioned samples into continent-based regions^11,20,21^. Using our novel genome sequences, we were able to increase the resolution of geographic analysis, and show that East Africa is the geographic source of HSV-2 worldwide diversity. However, we note the exact location and path the HSV-2 took upon leaving Africa is still unclear because of (i) the lack of samples from the Horn of Africa which is the likely source of human out-of-Africa migration, (ii) the sampling bias for viral genomes from North America, and (iii) human migration in the modern era which obscures ancient signal. Increased sampling across global regions could improve geographic signal of HSV-2 migration.

HSV-2 genome samples on the order of thousands of years should provide sufficient temporal signal to estimate the true clock rate of HSV-2. In our simulations, genomes sampled over a 47-year timespan can accurately calibrate a clock rate of 1.3 × 10^−6^ substitutions/site/year and faster. Hence HSV-2 genomes thousands of years old should have sufficient accumulation of signal to acutely infer a clock rate based on tip dates with a rate of 10^−8^ substitutions/site/year. The identification of ancient herpes virus sequences (including HSV-1) in mummified archeological specimens^28,33^ suggests the possibility of reconstructing ancient HSV-2 genomes that could be used to inform a tip-dated calibration for this virus and to reveal the ancient paths of HSV-2 transmission.

## Methods

### HSV-2 data set and sequence alignment

Clinical isolates were recovered from patients diagnosed with genital herpes at La Pitié Salpêtrière—Charles Foix University Hospital (Paris, France) who self-reported overseas origin. Due to the observational and retrospective nature of the study, the need for specific informed consent from individual patients was not required according to French law. All data generated in the present study had no impact on the routine standard clinical management of the patients. An informed consent to the treatment of personal data was acquired upon hospital admission from the patients or their legal representatives. HSV-2 isolates were used because of shortages of primary clinical sample material. Isolates were obtained by propagation in subconfluent Vero cell monolayers^34^). A limited number of passages was necessary for the generation of viral stocks.

We sequenced 65 HSV-2 samples collected 2012–2018 primarily from West and Central Africa. In brief, we extracted DNA from supernatant using the QIAmp Viral RNA Mini Kit (Qiagen, Hilden, Germany) and measured DNA content with a Qubit (Thermo Fischer Scientific, Waltham, MA, USA). HSV-2 copy numbers were determined using a quantitative PCR (qPCR) assay^8^. We then prepared dual indexed libraries using 1 ng DNA extract using the Nextera XT Library Preparation Kit and the Nextera XT Index Kit (Illumina, San Diego, CA, USA), which we pooled so individual libraries would all bring in about the same number of HSV-2 genome copies (according to the qPCR results). The pool was subjected to a double-round hybridization capture with a MYbaits Custom Target Enrichment Kit designed to cover the whole genomes of HSV-1 (NC_001806), HSV-2 (NC_001798), and varicella-zoster virus (VZV, NC_001348; *Human herpesvirus 3*) (Daicel Arbor Biosciences, Ann Arbor, MI, USA). For both rounds, we followed manufacturer’s instructions with the exception that only one-fourth of the recommended bait quantity was used. We sequenced the capture product on MiSeq and NextSeq 550 platforms (Illumina) using the MiSeq Reagent Kit v3 (600 cycles; Illumina) and NextSeq Mid Output Kit v2.5 (300 cycles; Illumina), generating a total of 61,926,628 reads.

Low quality bases and adapter sequences were removed from raw reads using Trimmomatic v0.36^35^ and overlapping reads were merged using ClipAndMerge v1.7.8^36^. We then mapped trimmed reads to the HSV-2 RefSeq genome (NC_001798) from which we had removed the TRL and TRS regions^37^. We finally sorted mapping files and removed duplicate reads using the SortSam and MarkDuplicates tools from Picard v2.7.1 (http://broadinstitute.github.io/picard/). We generated consensus sequences from the maps using Geneious Prime v2021.2.2^38^, calling bases at positions covered by at least 20 unique reads and for which more than 95% of the reads were in agreement.

Additionally, we downloaded all available HSV-2 sequences of length greater than 100kbp from GenBank in November 2019. Potentially engineered (*n* = 5) and dubious sequences (*n* = 10) were removed. HSV-2 sequences along with, ChHV (NC_023677) and HSV-1 (NC_001806) were aligned with MAFFT v7.307^39^. Regions with more than 10% of sequences containing gaps were removed with Geneious. Invariant sites were stripped from the alignment to produce a dataset that was computationally tractable for recombination analysis, resulting in an alignment of 25,809 variant sites of ChHV, HSV-1, and HSV-2 sequences. Recombinant fragments were inferred with RDP4^40^, using five recombination detection methods (RDP, GENECONV, MaxChi, BootScan, and SiScan) and validating recombination events identified by at least two methods. In sequences found to contain recombination, the recombinant fragments within these sequences were masked (with Ns) in the complete alignment (including variant and invariant sites) for subsequent phylogenetic analysis.

### Phylogenetic and initial temporal analysis

Subsequent analysis was done on only HSV-2 sequences with the year of sample collection resulting in a final HSV-2 dataset of 395 sequences, of 105,420 sites of which 5648 sites had variation. Sequences were sampled globally across 47 years, from 1971 to 2018 (Supplementary Data 1).

A maximum likelihood phylogeny for the HSV-2 dataset was made using IQtree2 v2.0.4^19^, with a GTR + F + R4 substitution model selected by model testing using ModelTesting with cAIC on the initial alignment^41^. To test uncertainty in the topology we used aBayes to estimate branch support^42^. We used TreeTime v0.7.6^22^ to perform molecular clock inference on HSV-2 using tip date calibrations. Default TreeTime parameters were used, except for fixing the root, using input branch lengths, and clock filter was set to 0 (no filtering). The root was fixed, as this rooting is supported by including ChHV as an outgroup and is consistent with previous studies^8^. Input topology and branch lengths were used to because joint optimization is not stable without this approach^43^. With the lack of clock like signal it is was not appropriate to filter based on clock variation.

### Likelihood of fixed clock rates with empirical data

The log likelihood was calculated for time trees with a range of fixed substitution rates using TreeTime. We used a fixed input tree, empirical sample dates, and no clock filter. The rates used were 10^−9^ substitutions/site/year to 10^−3^ substitutions/site/year, with a resolution of 8 rates distributed evenly across the log space across each order of magnitude. This distribution exceeds the range of previous clock rate estimates for simplexvirus and HSV-1^11,16^.

To validate the interpretation of the likelihood surface, we performed a similar process with a previously published dataset of 1,610 ebolavirus sequences from the 2014 to 2015 West African Ebola epidemic^23^, which has detectable temporal signal using tip-date calibrations. The Ebola dataset was analyzed with rates from 10^−1^ substitutions/site/year to 10^−5^ substitutions/site/year, with a resolution of three rates for each order of magnitude spread evenly across log space (Supplementary Fig. 2).

### Clock rate estimates of simulated data

In order to understand what clock rate would result in sufficient temporal signal to detect the molecular clock on the empirical HSV-2 dataset (i.e., range of sampling dates, sequence length, and tree topology), we simulated sequence data across a range of substitution rates, 10^−9^ to 10^−3^ substitutions/site/year with eight rates across each order of magnitude. Simulation was done with the nucleotide substitution model parameters inferred by IQtree2, under a GTR + F + Γ4 model on the HSV-2 dataset. We first inferred a time tree with a fixed clock rate based on our topology using TreeTime and then scaled this tree to substitutions/site with the clock rate. Sequences were simulated on the rescaled tree with Seq-Gen v1.3.4^44^. We performed 200 replicates of sequence evolution and subsequent analysis.

Branch lengths were estimated on the fixed empirical topology using the simulated sequences with IQtree2 using GTR + F + Γ_4_ model. Although the empirical tree inference was performed with free rate variation, we used gamma rate variation in the simulations and subsequent tree inference to maintain consistency between simulation, tree, and clock inference. The clock rate and *R*^2^ of the root-tip-regression of the simulated data were then estimated with TreeTime, using previous parameters.

The detection of temporal signal was assessed through different methods using R^45^. The rate at which there was a change in the relationship between the mean absolute value of estimated rate and the simulated rate was inferred by a linear regression break point analysis (R segmented v. 1.2-0).

The point at which the estimated rate reflected the empirical data, in that the estimated clock rate was negative, was determined by two cutoffs. The first cutoff method is the fastest rate at which more than 5% of estimated rates on simulated data were negative. The second cutoff is the fastest rate at which a negative estimate is as likely as a positive estimate, determined by binomial test (*p* < 0.05).

### Phylogeographic ancestral state reconstruction

We performed a migration analysis, which is an inference of transition between discrete geographic regions along branches of the phylogenetic tree, to reconstruct the ancestral geographic state. We used TreeTime migration with default parameters, empirical time trees built with different fixed clock rates and the region of sampling for each tip. Samples with country histories were partitioned into regions, based on the United Nations M49 Standard, into North Africa, West Africa, East Africa, Central Africa, Southern Africa, and countries from all other regions were grouped into non-African region. Six samples had no country of origin, but are known to be sampled from Africa, and were partitioned into a broad Africa, region unknown for phylogeography analysis. Additionally, four samples were from an unknown location and were indicated as missing in the migration analysis.

Robustness analysis of the phylogeographic reconstruction was done by repeating previous migration analysis with a merged West, Central, and Southern Africa region. Additional robustness analysis split the non-African region into worldwide subregions, and reconstruction was done with North Africa, West Africa, East Africa, Central Africa, Southern Africa, Africa region unknown, Eastern Europe, Southern Europe, Western Europe, Northern America, South America, Southeastern Asia, Western Asia, and missing.

The points of particular interest in the trees are first the age of the root, which represents the age of HSV-2 diversification, and second the age of the MRCA of the worldwide diversity within the worldwide lineage, which represents the maximum age for the dispersal of HSV-2 out-of-Africa, assuming an African origin for the lineage B. On the fixed topology, ancestral geographic reconstruction along the basal backbone of the worldwide lineage had majority of support for the African region that is the source of HSV-2 worldwide diversity which decreases until the majority of support is for the non-African region (Supplementary Figure 5). We defined the-out of-Africa age as the mean age of the nodes that define the backbone at the base of lineage B, weighted by a factor of one minus the absolute value of the difference of the support for source African region and support for non-African region. This factor accounts for the uncertainty which point on the maximum likelihood phylogeny that HSV-2 migrated out-of-Africa.

Age is reported in number of years ago, relative to 2018, the most recent sample date.

### Reporting summary

Further information on research design is available in the Nature Research Reporting Summary linked to this article.

## Supplementary information

Supplementary Information

Description of Additional Supplementary Files

Supplementary Data 1

Supplementary Data 2

Supplementary Data 3

Supplementary Data 4

Reporting Summary

## Data Availability

All sequence data generated in this study are made available in BioProject under accession code PRJEB48656. All accession numbers for data used in this manuscript can be found in Supplementary Data 1. Code for running simulation is available at 10.5281/zenodo.7054908.

## Code availability

The latest code for running simulation is available at https://github.com/jennifer-bio/HSV2, code used to run simulation for this manuscript can be referenced at 10.5281/zenodo.7054908.
