# Supplementary material for: Phylogeographic analysis reveals an ancient East African origin of human herpes simplex virus 2 dispersal out-of-Africa
Source: Nat Commun. 2022 Sep 17;13:5477. doi: 10.1038/s41467-022-33214-y (PMC9482657; doi:10.1038/s41467-022-33214-y)
Supplement: Supplementary file 1 — Supplementary Information [file 41467_2022_33214_MOESM1_ESM.pdf]

SUPPLEMENTARY INFORMATION:

Phylogeographic analysis reveals an ancient East African origin of human herpes simplexvirus 2 dispersal out-of-Africa

Authors:
Jennifer L. Havens, Sébastien Calvignac-Spencer, Kevin Merkel, Sonia Burrel, David Boutolleau, Joel O. Wertheim

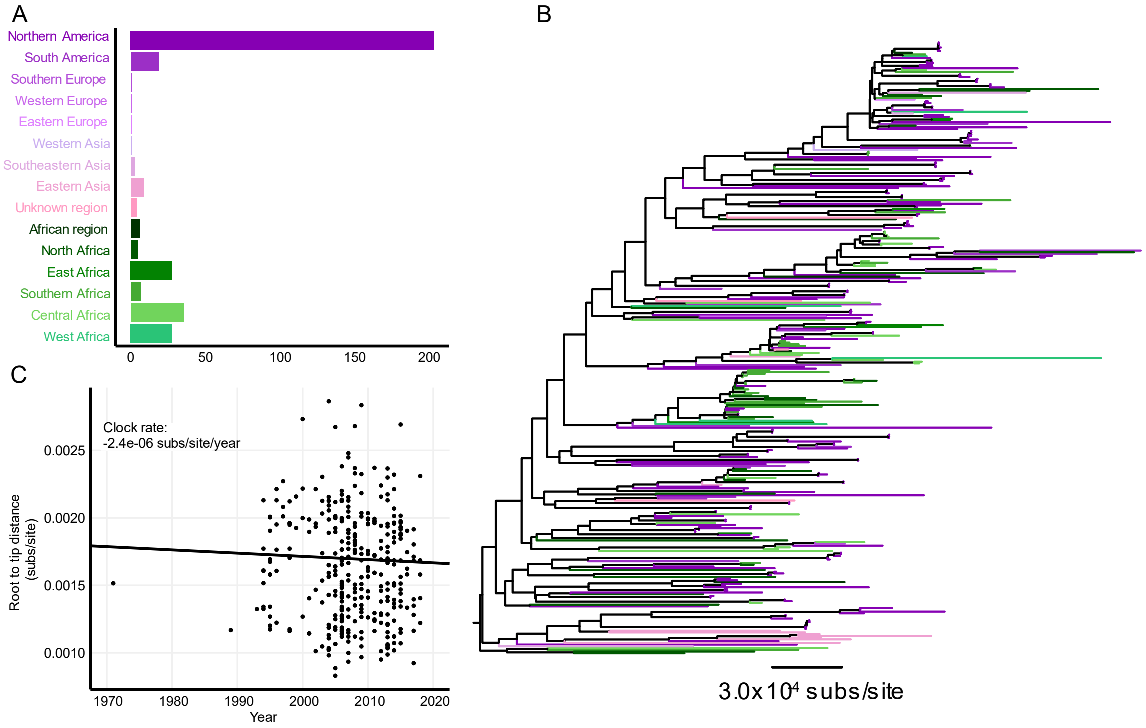

Fig. S1. Phylogenetic analysis of subset of HSV-2 worldwide lineage. (A) HSV-2 dataset of 353 sequences in out of Africa clade of worldwide lineage, by number of samples from each region. (B) ML phylogenetic tree inferred with GTR+F+R4 model of sequences described in panel A. Color indicates region of sampling for tip. (C) Root to tip distance of ML tree verse sampling year, with regression estimate of clock rate of -2.4×10^{-6} substitutions/site/year.

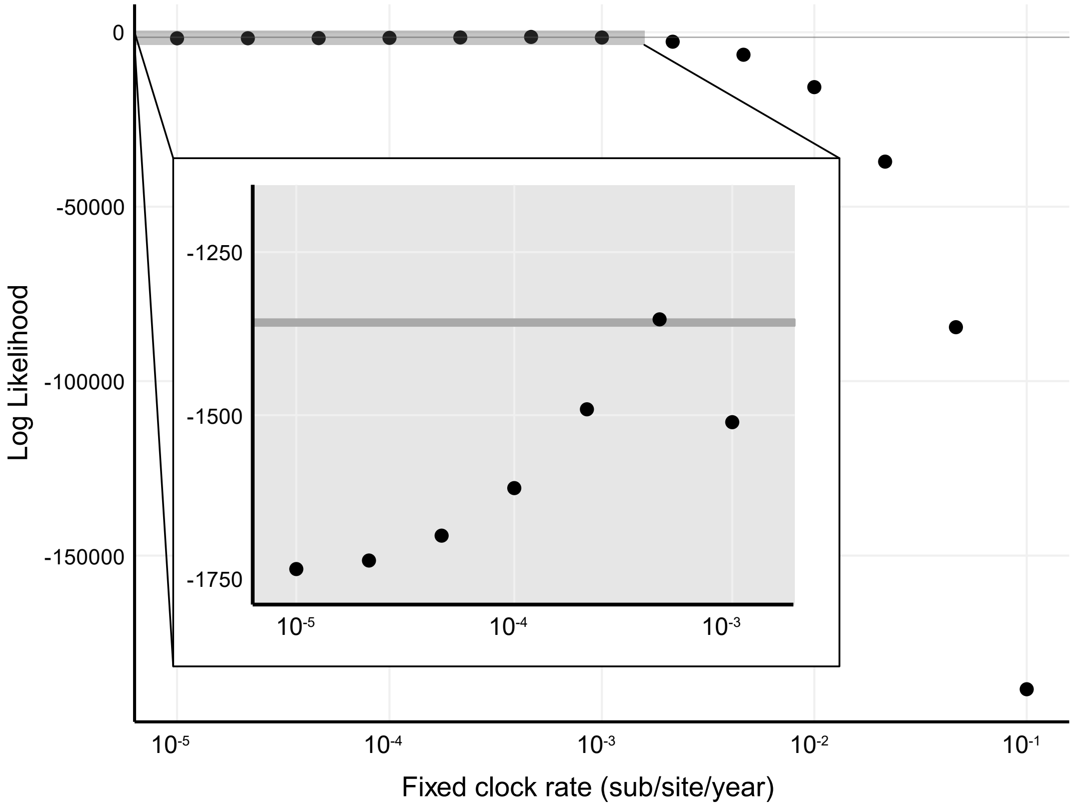

Fig. S2. Shape of likelihood surface for clock rate of ebolavirus. Log likelihood of ebolavirus time tree at fixed clock rates estimated using input ML tree (1) of 1610 Ebola genomes, black line is maximum estimated likelihood, grey shaded box is the area shown in insert. (Insert) Black line is maximum estimated likelihood at rate 4.6×10^{-4} substitutions/site/year, dark grey box is range from maximum estimated likelihood to 10 points below.

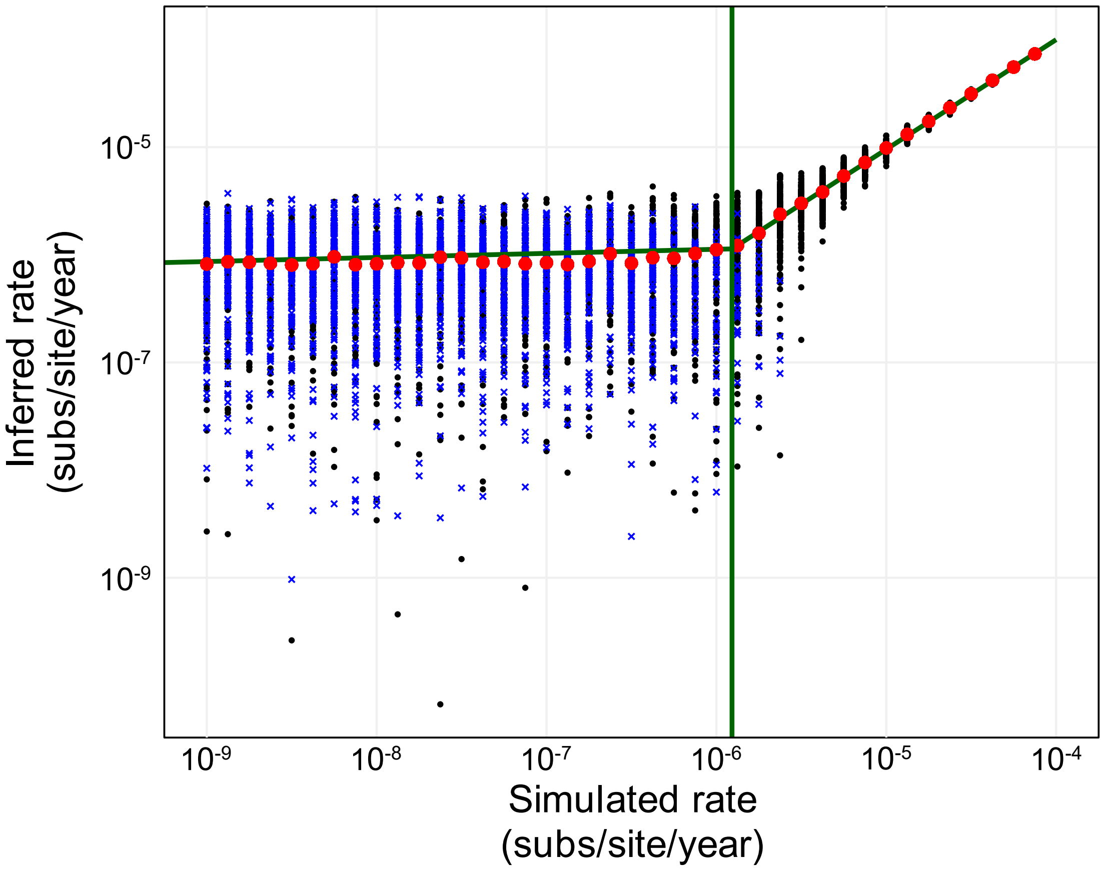

Fig. S3. Breakpoint analysis of clock rate inference on simulated data. Estimates of rate for a fixed input tree with simulated sequences and empirical dates. Black dot represents estimates of positive rates, while blue 'x' represent the absolute value of estimates of negative rates, and the mean of absolute value for each fixed rate is indicated by red dot. Sloped green segments represent break point regression of absolute value of estimated rates. Vertical green line represents break point at 1.2×10^{-6} substitutions/site/year (R segmented). 200 replicates were generated under GTR+Γ_4 model with fixed rates 1×10^{-9} to 1×10^{-4} substitutions/site/year.

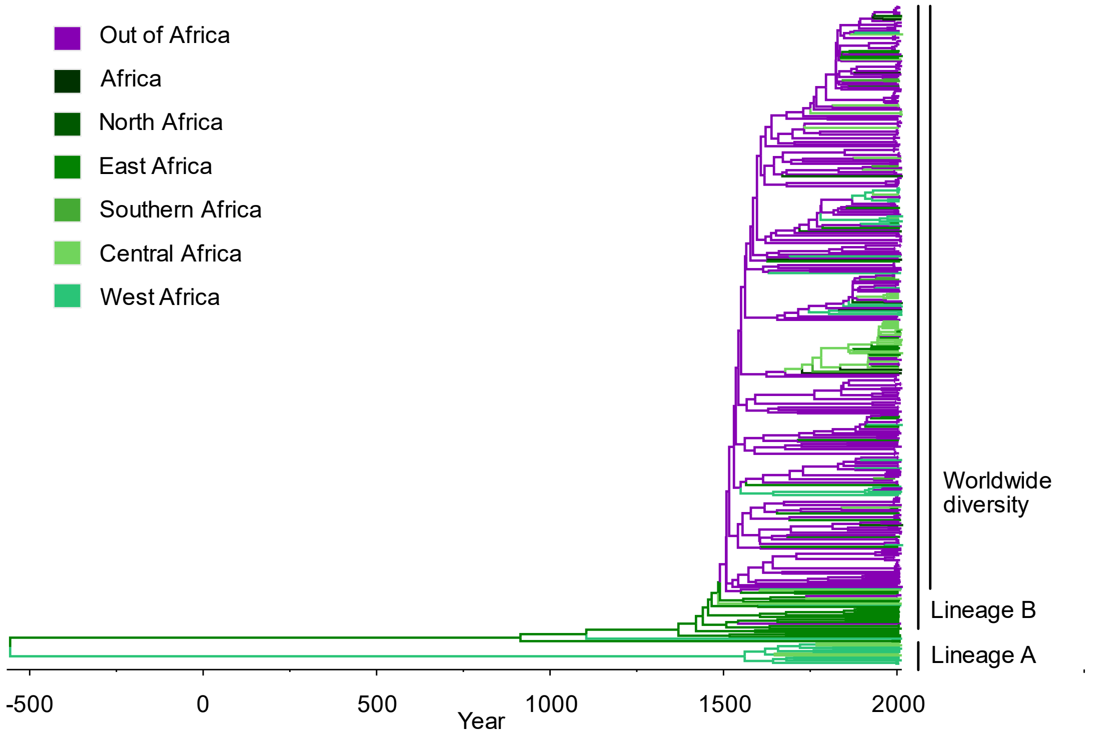

Fig. S4.
Phylogeography of HSV-2 supports East Africa as origin of out-of-Africa dispersal. Time tree inferred with the fastest rate supported by any clock analysis, 2.4×10^{-6} substitutions/site/year. ML tree topology scaled to time tree, colored by plurality of support in mugration analysis for each region. Time scale represents the calendar year; negative years are BCE.

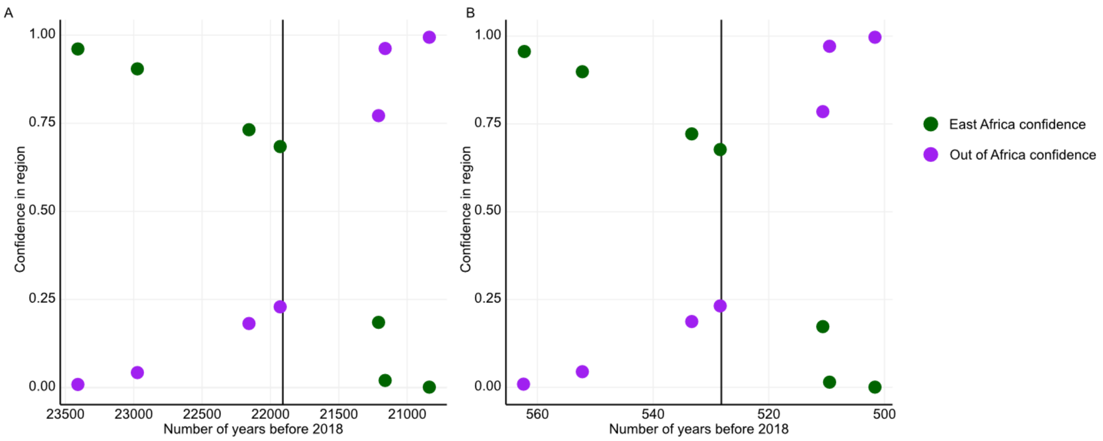

Fig. S5. Internal nodes in backbone of tree around out of Africa migration (within the worldwide lineage). Confidence in East Africa placement (green) and out of Africa placement (purple), by number of years before 2018, for time trees with rate $5.8x10^{-8}$ (A) and $2.4x10^{-6}$ (B) substitutions/site/year. Vertical bar indicates average age of nodes with ages weighted by 1-absolute value(East Africa confidence - out of Africa confidence).
