# Supplementary material for: Phylogeographic analysis reveals an ancient East African origin of human herpes simplex virus 2 dispersal out-of-Africa
Source: Nat Commun. 2022 Sep 17;13:5477. doi: 10.1038/s41467-022-33214-y (PMC9482657; doi:10.1038/s41467-022-33214-y)
Supplement: Supplementary file 2 — Description of Additional Supplementary Files [file 41467_2022_33214_MOESM2_ESM.pdf]

Description of Additional Supplementary Files

File Name: Supplementary Data 1
Description: Sample metadata table with sequence information.

File Name: Supplementary Data 2
Description: Nexus formatted treefile of empirical HSV-2 dataset, visualized in Fig. 1. ML tree of 395 HSV-2 sequences estimated with GTR+F+R4. The tree displayed is midpoint rooted, though the identical rooting orientation is recovered using the closely related chimpanzee simplexvirus. Node labels indicate aBayes support.

File Name: Supplementary Data 3
Description: Nexus formatted time trees inferred using with fixed evolutionary rates. Associated summary of ages and phylogeographic source in Table 1.

File Name: Supplementary Data 4
Description: Phylogeographic analysis summary table with age of root and out-of-Africa dispersal across clock rates for robustness analysis of geographic partitioning.
