# Supplementary material for: Phylogeographic analysis reveals an ancient East African origin of human herpes simplex virus 2 dispersal out-of-Africa
Source: Nat Commun. 2022 Sep 17;13:5477. doi: 10.1038/s41467-022-33214-y (PMC9482657; doi:10.1038/s41467-022-33214-y)
Supplement: Supplementary file 7 — Reporting Summary [file 41467_2022_33214_MOESM7_ESM.pdf]

nature portfolio

Corresponding author(s): Jennifer L Havens

Last updated by author(s): 2022-09-02

Reporting Summary

Nature Portfolio wishes to improve the reproducibility of the work that we publish. This form provides structure for consistency and transparency in reporting. For further information on Nature Portfolio policies, see our Editorial Policies and the Editorial Policy Checklist.

Statistics

For all statistical analyses, confirm that the following items are present in the figure legend, table legend, main text, or Methods section.

n/a	Confirmed	
☐	☒	The exact sample size (*n*) for each experimental group/condition, given as a discrete number and unit of measurement
☐	☒	A statement on whether measurements were taken from distinct samples or whether the same sample was measured repeatedly
☐	☒	The statistical test(s) used AND whether they are one- or two-sided<br>*Only common tests should be described solely by name; describe more complex techniques in the Methods section.*
☒	☐	A description of all covariates tested
☒	☐	A description of any assumptions or corrections, such as tests of normality and adjustment for multiple comparisons
☐	☒	A full description of the statistical parameters including central tendency (e.g. means) or other basic estimates (e.g. regression coefficient) AND variation (e.g. standard deviation) or associated estimates of uncertainty (e.g. confidence intervals)
☐	☒	For null hypothesis testing, the test statistic (e.g. *F*, *t*, *r*) with confidence intervals, effect sizes, degrees of freedom and *P* value noted<br>*Give P values as exact values whenever suitable.*
☒	☐	For Bayesian analysis, information on the choice of priors and Markov chain Monte Carlo settings
☒	☐	For hierarchical and complex designs, identification of the appropriate level for tests and full reporting of outcomes
☒	☐	Estimates of effect sizes (e.g. Cohen's *d*, Pearson's *r*), indicating how they were calculated

Our web collection on statistics for biologists contains articles on many of the points above.

Software and code

Policy information about availability of computer code

Data collection	No software was used for data collection
Data analysis	Data analysis was performed using Trimmomatic (0.36), ClipAndMerge (1.7.8), Picard (2.7.1), Geneious Prime (2021.2.2), MAFFT (v7.307), RDP4, IQtree2 (v2.0.4) , TreeTime (v0.7.6),Seq-Gen (v1.3.4), R (v3.6.0), R segmented (v1.2-0), code for running simulation available at https://github.com/jennifer-bio/HSV2

For manuscripts utilizing custom algorithms or software that are central to the research but not yet described in published literature, software must be made available to editors and reviewers. We strongly encourage code deposition in a community repository (e.g. GitHub). See the Nature Portfolio guidelines for submitting code & software for further information.

Data

Policy information about availability of data

All manuscripts must include a data availability statement. This statement should provide the following information, where applicable:

- Accession codes, unique identifiers, or web links for publicly available datasets
- A description of any restrictions on data availability
- For clinical datasets or third party data, please ensure that the statement adheres to our policy

All sequence data generated were made available under bioproject PRJEB48656. Accession numbers for all sequences used can be found in supplementary dataset 1.

Human research participants

Policy information about studies involving human research participants and Sex and Gender in Research.

Reporting on sex and gender	N/A
Population characteristics	N/A
Recruitment	N/A
Ethics oversight	N/A

Note that full information on the approval of the study protocol must also be provided in the manuscript.

Field-specific reporting

Please select the one below that is the best fit for your research. If you are not sure, read the appropriate sections before making your selection.

☐ Life sciences ☐ Behavioural & social sciences ☒ Ecological, evolutionary & environmental sciences

For a reference copy of the document with all sections, see nature.com/documents/nr-reporting-summary-flat.pdf

Ecological, evolutionary & environmental sciences study design

All studies must disclose on these points even when the disclosure is negative.

Study description	Clinical isolates were recovered from patients diagnosed with genital herpes at La Pitié Salpêtrière – Charles Foix University Hospital (Paris, France) who self-reported overseas origin; isolates were propagated and sequenced.
Research sample	HSV-2 virus were sampled from humans.
Sampling strategy	Convenience sampling from patients who self-reported origins from overseas
Data collection	Clinical isolates were from patients diagnosed with genital herpes.
Timing and spatial scale	Primary samples were collected at La Pitié Salpêtrière – Charles Foix University Hospital (Paris, France) from 2013 - 2018. Patients self-reported origins from overseas.
Data exclusions	No exclusions.
Reproducibility	N/A
Randomization	No randomization, it is not relevant as this is an observational study.
Blinding	No blinding, it is not relevant as this is an observational study.

Did the study involve field work? ☐ Yes ☒ No

Reporting for specific materials, systems and methods

We require information from authors about some types of materials, experimental systems and methods used in many studies. Here, indicate whether each material, system or method listed is relevant to your study. If you are not sure if a list item applies to your research, read the appropriate section before selecting a response.

Materials & experimental systems

n/a	Involved in the study	
☒	☐	Antibodies
☒	☐	Eukaryotic cell lines
☒	☐	Palaeontology and archaeology
☒	☐	Animals and other organisms
☒	☐	Clinical data
☒	☐	Dual use research of concern

Methods

n/a	Involved in the study	
☒	☐	ChIP-seq
☒	☐	Flow cytometry
☒	☐	MRI-based neuroimaging
